# The Role of Pharmacovigilance Database in Identifying Antibiotic Resistance and Inappropriate Use: An Analysis of VigiBase Reports From Lower‐Middle‐Income Countries

**DOI:** 10.1002/pds.70404

**Published:** 2026-06-08

**Authors:** Hager Saleh, Joseph Mitchell, Cecilia Stålsby Lundborg, Megha Sharma

**Affiliations:** ^1^ Department of Global Public Health, Health Systems and Policy Karolinska Institutet Stockholm Sweden; ^2^ Department of Pharmacology Ruxmaniben Deepchand Gardi Medical College Ujjain India

**Keywords:** antibiotics, antimicrobial resistance, AWaRe classification, drug ineffectiveness, individual case safety reports, lower‐middle‐income countries, MedDRA, off‐label use, pharmacovigilance, RIOLE

## Abstract

**Background:**

Digital databases such as pharmacovigilance (PV) databases could provide unique opportunities to monitor trends in suspected antibiotic resistance, ineffectiveness, and misuse, extending beyond their traditional role of tracking adverse drug reactions (ADRs). This approach is potentially valuable globally but particularly advantageous in lower‐middle‐income countries (LMICs) where formal resistance surveillance systems are often insufficiently developed. Leveraging PV data could help generate early signals of resistance and inappropriate antibiotic use and support antimicrobial stewardship in resource‐constrained settings.

**Objectives:**

To explore the potential use of PV databases in monitoring suspected antibiotic resistance trends and inappropriate use in LMICs.

**Methods:**

A retrospective cross‐sectional study was conducted using VigiBase. Data were extracted in October 2024 from inception to January 1, 2024. Reports involving antibacterials for systemic use, Anatomical Therapeutic Chemical (ATC) codes J01 and J04 from LMICs were included. Selected Medical Dictionary for Regulatory Activities (MedDRA) preferred terms were mapped according to RIOLE classification to the “resistance,” “ineffectiveness,” “off‐label use,” and “error” categories to identify reporting patterns. Descriptive statistics were used to summarize reports' characteristics, and associations between categorical variables were examined using chi‐squared tests.

**Results:**

A total of 1570 ICSRs from 37 LMICs were identified, yielding 2958 drug–adverse event pairs, with reporting increasing markedly after 2016. The “off‐label use” (38.6%) and “ineffectiveness” (37.0%) were the dominant RIOLE categories, driven mainly by the preferred terms (PTs) of *Off‐label use* (795; 26.4%) and *Drug ineffective* (751; 25.4%). *Resistance‐related* PTs accounted for 12.7% of pairs, most frequently *Drug resistance* (210; 7.0%) and *Pathogen resistance* (132; 4.5%), while “error” category (11.7%) was led by *Product use issue* (60; 2.0%) and *Medication error* (44; 1.5%). *Watch* antibiotics predominated, especially azithromycin, ceftriaxone, and meropenem, with significant associations observed between RIOLE categories and age, reporter type, ATC class, reaction outcome, AWaRe category, and WHO region.

**Conclusions:**

These findings demonstrate that PV databases can provide valuable insights into suspected antibiotic resistance and inappropriate use patterns in LMICs, supporting their potential role as additional data sources in antimicrobial stewardship.

## Key Points

1. Pharmacovigilance databases can capture reports of suspected antibiotic resistance and inappropriate use
2. Strengthening reporting quality and promoting the use of AMR‐related reporting terms among healthcare professionals could strengthen pharmacovigilance as a complementary tool for AMR surveillance, especially where laboratory capacity is limited.
3. Given the challenge of underreporting of adverse drug reactions, the presented results of hundreds of reports may be the tip of the iceberg, contributing to increased resistance worldwide.
4. Reports reflecting suspected treatment failure and inappropriate use (resistance and innefectivness categories, RI) and those “off‐label use” and “error” (OLE) require distinct interventions.
5. The high proportion of off‐label use reports involving *Watch* and *Reserve* antibiotics might indicate widespread inappropriate prescribing of the most critically important antibiotics, where such misuse accelerates resistance.

## Plain Language Summary

Antibiotics sometimes fail to work as expected, either because the bacteria are resistant, the wrong antibiotic was chosen, or the medicine was used incorrectly. In this study, we analyzed reports from lower‐ middle‐income countries (LMICs) submitted to the World Health Organization's global safety database (VigiBase) to understand why antibiotic treatments go wrong. We found that most reports were related to antibiotics being used for the wrong indication or not working as intended, while only a smaller number described confirmed resistance. These patterns likely reflect challenges in LMICs' health systems, such as limited access to diagnostic tests and the need to make treatment decisions based on symptoms alone. The study also shows that safety reporting systems, originally designed to detect side effects, can provide important early warnings about possible antimicrobial resistance, especially in places where laboratory testing is limited. Strengthening antibiotic prescribing practices, improving how health workers report treatment failures, and integrating pharmacovigilance data into national antimicrobial resistance programs could help countries identify problems sooner and support safer, more effective use of antibiotics.

**Abbreviations**
ADRs: adverse drug reactions
AE: adverse event
AMR: antimicrobial resistance
ATC: anatomical therapeutic chemical
AWaRe: WHO's antibiotic classification *Access*, *Watch*, *Reserve*
J01: ATC code of antibacterial for systematic use
J04: ATC code of antimycobacterial
MedDRA: medical dictionary for regulatory activities

## 1 Introduction

Antibiotics have transformed modern medicine since the introduction of salvarsan, the first antibiotic in 1910, extending the average human lifetime by 23 years, alongside broader public health improvements [1]. The discovery of penicillin in 1928 triggered a golden period of antibiotic discovery, which peaked in the 1950s [1]. However, a drop in new antibiotic development and the increase of drug‐resistant bacteria have contributed to the current antimicrobial resistance (AMR) dilemma [1].

AMR is driven in part by the inappropriate use or misuse of antibiotics in human, animal health and in agriculture and aquaculture contributing to the spread of resistance genes, creating a silent pandemic [2]. Moreover, antibiotic misuse puts patients at risk for adverse drug reactions (ADRs), temporary symptom improvement without eradication of the underlying infection, and the rise of drug‐resistant microorganisms [3].

The consequences of AMR include treatment failure, increased morbidity and mortality. The burden of AMR has higher significance in low‐ and middle‐income countries due to various factors, for example, limited access to diagnostics, increased burden of infectious diseases, improper prescribing, negative attitude of healthcare professionals and the population, and the unregulated or underregulated use of medicines [2, 3].

Traditional AMR surveillance largely depends on microbiological testing and laboratory networks, which are often expensive and resource‐intensive [4]. Many LMICs face major gaps in structured surveillance systems and have limited laboratory capacity [4]. In response, there is growing interest in alternative, complementary, and more cost‐effective surveillance methods, which include data‐driven approaches or other nonlaboratory sources [5].

Pharmacovigilance (PV) is traditionally used to monitor ADRs, but its potential use in tracking antibiotic resistance and inappropriate use is emerging [6, 7, 8, 9, 10, 11]. PV databases play a key role in global drug safety monitoring. One such is VigiBase, the World Health Organization's (WHO) global database of ADRs for medications and vaccines [12]. VigiBase contains more than 40 million reports from over 180 countries participating in the WHO Program for International Drug Monitoring (PIDM) [12]. It contains reports on suspected ADRs that include resistance‐related terms for example, *Drug ineffective*, *Pathogen resistance*, *Off‐label use* [6, 11]. Some studies have demonstrated that PV data might serve as an early warning tool for AMR trends [6, 7, 10, 11, 13]. Despite this emerging potential, only a limited number of studies have explored how PV data can be systematically applied for AMR surveillance, in LMICs [6, 7, 10, 11, 13]. Building on this gap, the present study analyzes ADR reports from VigiBase using selected Medical Dictionary for Regulatory Activities (MedDRA) preferred terms (PTs) to assess antibiotic resistance and inappropriate use in LMICs.

The present study aims to explore how PV data can be used to identify potential cases of antibiotic resistance and inappropriate use in LMICs. Specifically, it sought to identify relevant MedDRA terms related to resistance and misuse on reports in LMICs, analyze these ADR reports, and provide evidence on the potential contribution into how PV data can contribute to AMR surveillance and stewardship efforts.

## 2 Materials and Methods

### 2.1 Identification of AMR‐Relevant PTs

MedDRA is standardized international medical terminology used by regulators and the pharmaceutical industry across all stages of the regulatory process, from pre‐ to postmarketing, for data entry, retrieval, evaluation, and presentation [14]. Preferred Terms (PTs) represent a distinct medical concept (e.g., a symptom, diagnosis, or procedure). Analyzes were conducted at the PT level where PTs are used to report ADRs [15]. For broader clinical interpretation, PTs were also grouped according to their corresponding System Organ Classes (SOCs), which aggregate related terms based on etiology, anatomical site, pathophysiology, or purpose [14].

A set of MedDRA PTs relevant to AMR, therapeutic ineffectiveness, off‐label use, and medication errors was established through a structured process. Firstly, previously reported PTs in the literature were reviewed [6, 7]. Secondly, the identified terms were verified against the MedDRA dictionary, version 26.0, and each was linked to its corresponding MedDRA code [15]. Thirdly, additional potentially relevant PTs were identified by manually reviewing terms under the following MedDRA SOCs: 1. *Infections and infestations*, 2. *General disorders and administration site conditions*, 3. *Injury, poisoning, and procedural complications*, and 4. *Investigations*. Finally, the PTs were classified into one of four categories: “resistance” (R), “ineffectiveness” (I), “off‐label use” (OL), and “error” of use (E), corresponding to the RIOLE classification framework [6]. The complete list of PTs, MedDRA codes, SOCs, RIOLE categories, source of the term identification is presented in the Table S1.

### 2.2 Selection of Countries

Countries were selected using a two‐step process. First, lower‐middle‐income economies (gross national income per capita USD 1136–4465) were identified based on the 2023 World Bank classification, as published on August 30, 2023 [16]. Second, from these LMICs, those that were members (full or associate) of the WHO PIDM were included, based on Uppsala Monitoring Center (UMC)'s official member list (last modified on January 25, 2023, checked on August 30, 2023) [17] as only member countries actively submitting reports to UMC were eligible, Figure 2.

### 2.3 Data Source and Extraction

Data was obtained from VigiBase, maintained by UMC. The search was performed on a frozen, de‐duplicated dataset [18] with data from inception to cut‐off date of January 1, 2024.

The search strategy was as follows:

- *Substances:* Antibacterials with Anatomical Therapeutic Chemical (ATC) codes J01 (antibacterial for systemic use) and J04 (antimycobacterial) [19], including fixed‐combination products.
- *Drug reported role:* Antibiotic as suspected or interacting.
- *ADR:* Antibiotic with adverse event of the 69 MedDRA PTs of interest which are described above.
- *Countries:* Only LMICs are members of the WHO PIDM.

**FIGURE 1:**
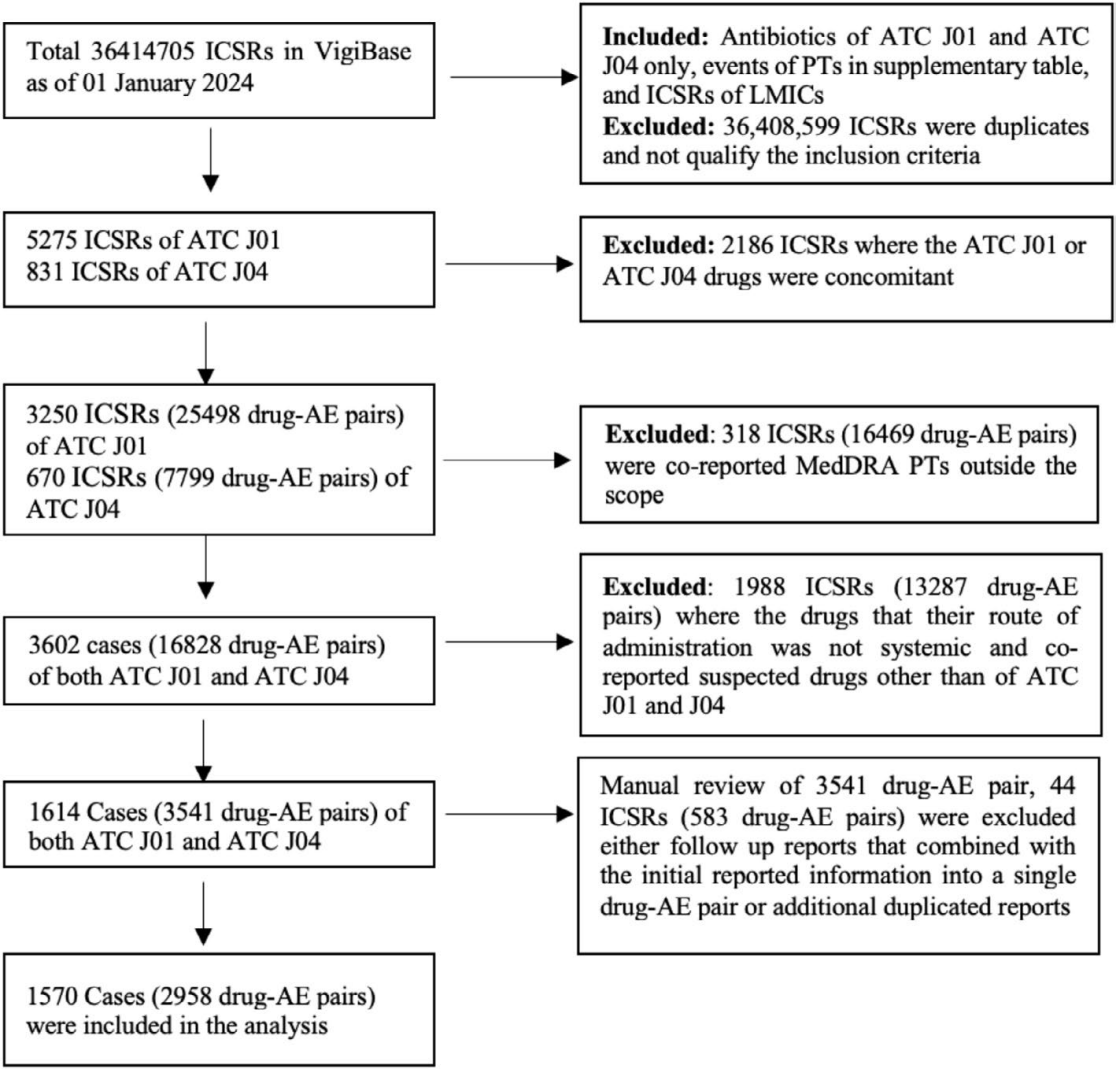
Flowchart of reports selection process from VigiBase for inclusion in the analysis. AE: Adverse Event. ATC: Anatomical Therapeutic Chemical. drug‐AE pair: Since each ICSR may include multiple drugs and multiple reported events, the drug–event pair is where each pair represents one suspected/interacted drug reported with one adverse event within an ICSR. ICSRs: Individual Case Safety Reports. J01: ATC code of antibacterial for systematic use. J04: ATC code of antimycobacterial. PTs: Preferred terms.

The process of the individual case safety reports (ICSRs) inclusion and exclusion is shown in Figure 1.

The detailed case‐level information was extracted including:

- Reports identification (unique number identifying each case report).
- Reporting date (date when the report was first entered in VigiBase) and reporting updated date (the last time the same report was entered in VigiBase for example, due to an update). These dates were used to identify the initial and follow‐up reports that were later combined.
- Continent of the primary source according to the United Nations and the WHO region of the primary source according to the WHO.
- The seriousness (seriousness criteria of the case including; caused or prolonged hospitalization, congenital anomaly, disabling, life‐threatening, and/or death).
- The type of report (spontaneous, report from literature, special monitoring, or other).
- The reporter (the reporter type was grouped by the researchers to be reports from healthcare professional or reports from nonhealthcare professional).
- Sex and the age group of patients (the age group of patients at time of onset of reaction/event).
- Drug name, drug role (suspect or interacting), ATC (J01 or J04), route of administration, indication (reason for drug use).
- MedDRA PT name of the reported adverse event and its outcome (the outcome of the reaction/event includes recovered/resolved, recovering/resolving, not recovered/not resolved, fatal, or unknown).

To ensure data privacy, the case narratives were not provided and the extracted data were presented at WHO regions [20, 21, 22, 23, 24, 25] and continent levels [26], without disclosing country specific counts.

### 2.4 Ethical Consideration

Ethical approval was not required for this study, as all data were fully anonymized prior to entry into VigiBase. All reports were coded and stored without personal identifiers, in accordance with established data protection and confidentiality standards.

### 2.5 Data Classification and Analysis

Antibiotics were classified according to both the WHO AWaRe framework (*Access*, *Watch*, and *Reserve* categories) [27] and the ATC classification system [19]. Microsoft Excel was used to manually review the cases where the initial case and its follow‐up are combined together to ensure the PT is counted once for each case. Since each ICSR may include multiple drugs and multiple reported events, the drug–event pair is where each pair represents one suspected/interacted drug reported with one adverse event within an ICSR. Descriptive statistics were applied to summarize the number and distribution of cases and reports by PT, drug class, WHO region, and reporting year. Chi‐squared (*χ* ^2^) tests were used to examine associations between the RIOLE categories and key variables such as gender, reaction outcome, AWaRe classification, reporter type, ATC class, and WHO region. Variables that were not reported were excluded from the *χ* ^2^ tests, and Cramer's V was calculated to quantify effect size. All analyzes were performed using R statistical software, version 4.3.3.

## 3 Results

**FIGURE 2:**
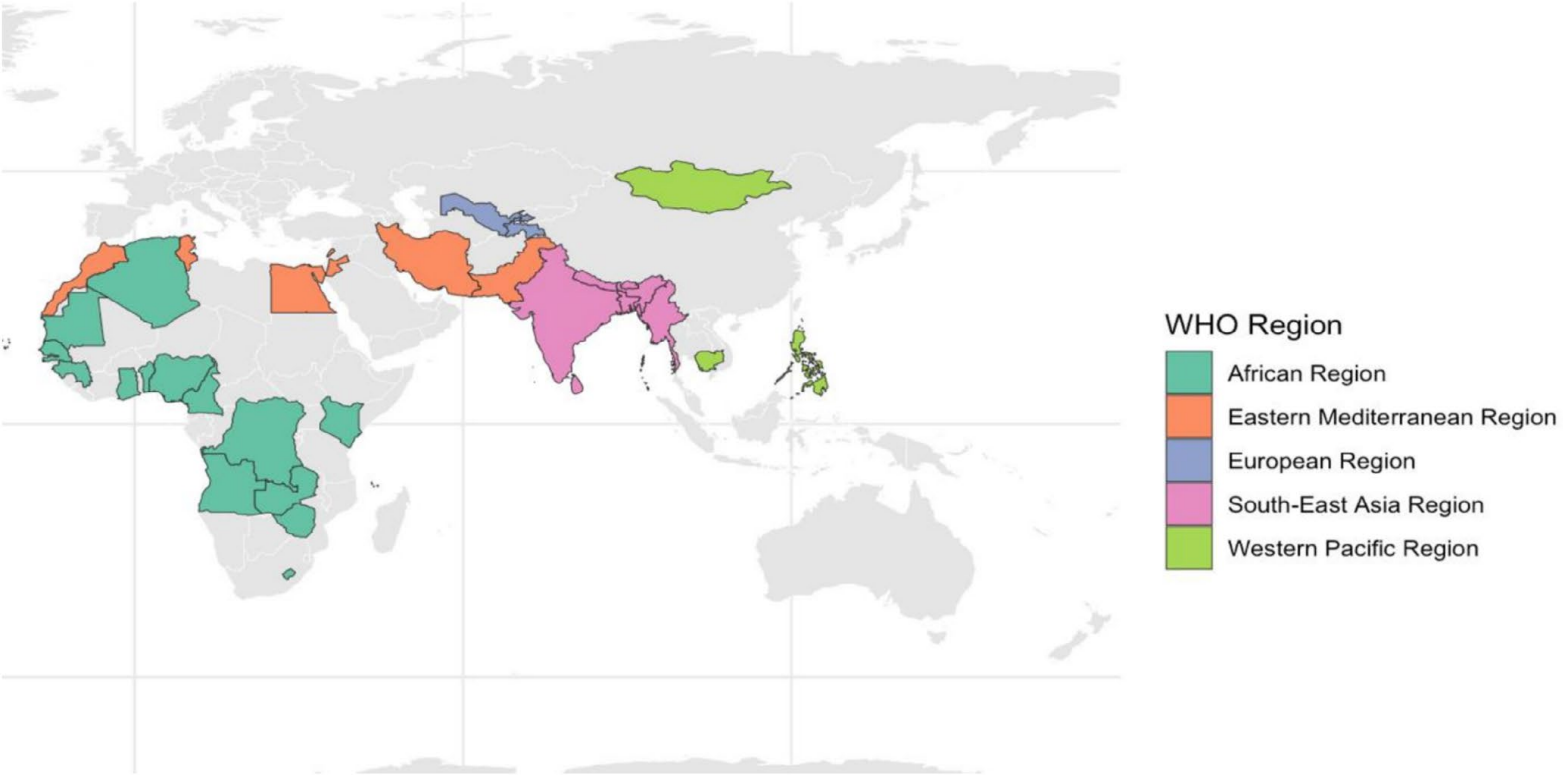
Geographic distribution of the LMICs included in the analysis that are members of the WHO PIDM included in the analysis. *LMICs not shown on the map indicate that no reports were retrieved from those countries. This map was generated using R; throughout the manuscript, data are presented at the group level to ensure privacy*.

**FIGURE 3:**
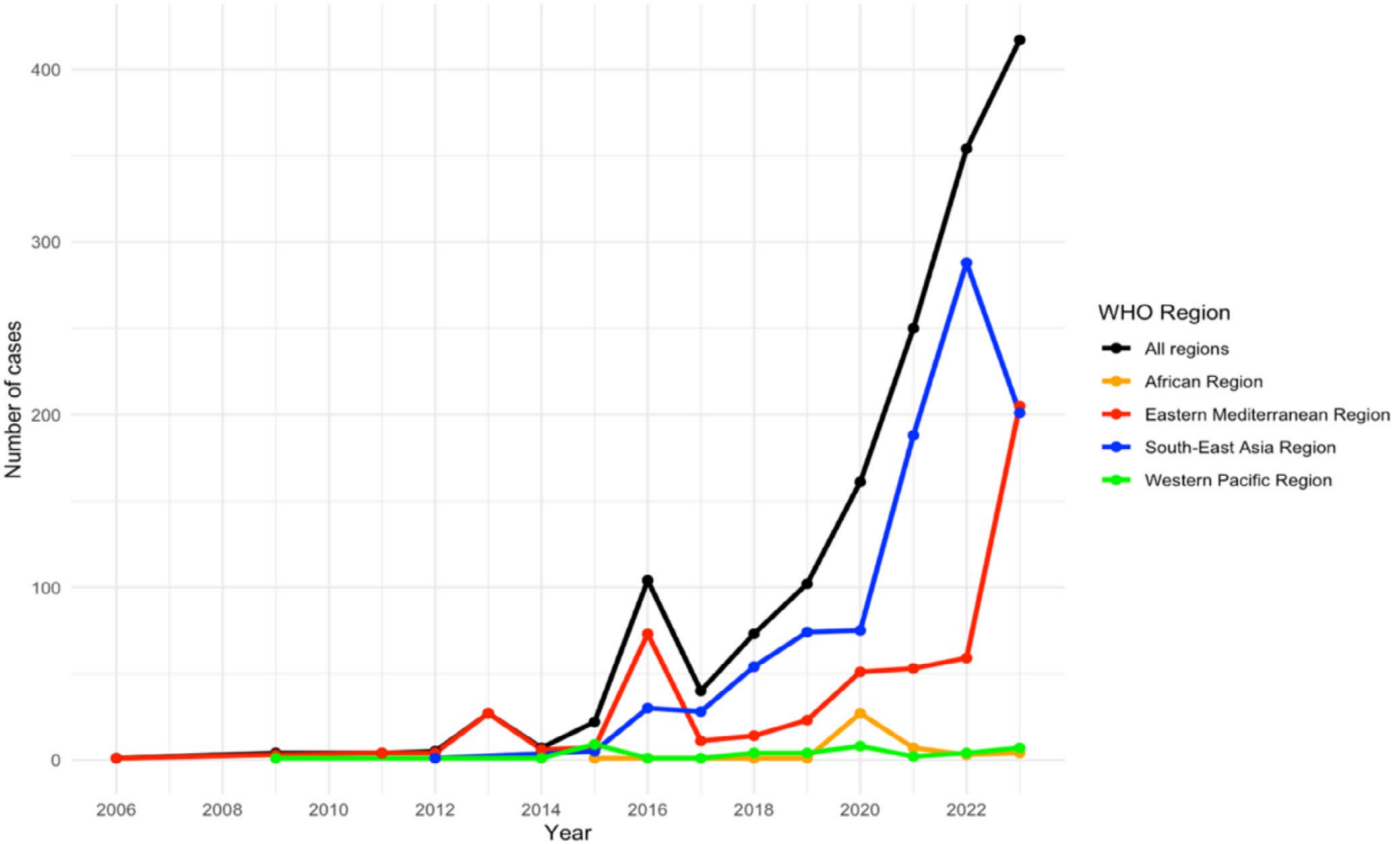
Temporal distribution of extracted cases across WHO Regions.

A total of 68 PTs were included in the data analysis, of which 37 were co‐reported relevant PTs outside the identified list described above, the full list shown in the Table S1. Retrieved cases were from 37 LMICs (Figure 2). The first eligible report meeting the study criteria was recorded in 2006. The total number of cases increased markedly over time, with a rise observed after 2016. The number of annual cases from all WHO regions showed a consistent upward trend, indicating growing reporting activity of the reporting of the antibiotic resistance and inappropriate use‐related PTs in VigiBase (Figure 3).

A total of 1570 ICSRs corresponding to 2958 drug‐AEs pairs reported were included in the analysis. Most cases occurred among adults aged 18–44 years (442; 28.2%) and 45–64 years (278; 17.7%), while (476; 30.3%) cases had unknown or unreported age. Most reports were submitted by healthcare professionals (1490; 94.9%) and spontaneous reports predominated (1030; 65.6%). More than half of the drug‐AE pairs were classified as serious (1572; 53.1%), most frequently due to caused/prolonged hospitalization or death (604; 20.4% and 423; 14.3%, respectively). By geographical distribution, most reports originated from the South‐East Asia Region (943; 60.1%) and the Eastern Mediterranean Region (541; 34.5%), (Tables 1 and 2).

**TABLE 1:** Characteristics of the individual cases.

	ICSRs, 1570 (100%) *n* (%)
Age group	
0–27 days	41 (2.6%)
28 days to 23 months	55 (3.5%)
2–11 years	93 (5.9%)
12–17 years	58 (3.7%)
18–44 years	442 (28.2%)
45–64 years	278 (17.7%)
65–74 years	79 (5.0%)
≥ 75 years	48 (3.1%)
Unknown/not reported	476 (30.3%)
Sex	
Male	789 (50.3%)
Female	480 (30.6%)
Unknown/not reported	301 (19.2%)
Reporter	
Healthcare professional	1490 (94.9%)
Nonhealthcare professional	76 (4.8%)
Unknown/not reported	4 (0.3%)
Report type	
Spontaneous	1030 (65.6%)
Report from medical/scientific literature	322 (20.0%)
Other	218 (13.9%)
Fatal case^a^	
Yes	218 (13.9%)
WHO region	
South–East Asia Region	943 (60.1%)
Eastern Mediterranean Region	541 (34.5%)
African Region	44 (2.8%)
Western Pacific Region	42 (2.7%)
Continent	
Asia	1161 (73.9%)
Africa	409 (26.1%)

Abbreviation: ICSRs: Individual Case Safety Reports.

^a^ A fatal case is if the case marked as fatal or has a seriousness criterion of death or the outcome of one of its events were reported as fatal.

**TABLE 2:** Characteristics of the drug‐AE pairs.

	Drug‐AE pairs, (total = 2958; 100%) *n* (%)
Drug role	
Suspect	2984 (99.7%)
Interacting	10 (0.3%)
Serious	
Yes	1572 (53.1%)
No	1386 (46.9%)
Seriousness criteria of serious events^a^	
Caused/prolonged hospitalization	604 (20.4%)
Disabling/incapacitating	24 (0.8%)
Life threatening	129 (4.4%)
Death	423 (14.3%)
Other	1097 (37.1%)
Unknown/not reported	19 (0.6%)
Mean drug‐AE pairs per ICSRs (±SD)	1.88 (±2.00)
Adverse event duration, days, mean (±SD)	7.29 (±16.40)

Abbreviations: Drug‐AE pair: Since each ICSR may include multiple drugs and multiple reported events, the drug–event pair is where each pair represents one suspected/interacted drug reported with one adverse event within an ICSR. ICSRs: Individual Case Safety Reports. SD: Standard deviation.

^a^ One serious event may fulfill more than one seriousness criterion; therefore, the total count of seriousness criteria exceeds 1572 events classified as serious (i.e., “Yes” for the Serious variable).

**FIGURE 4:**
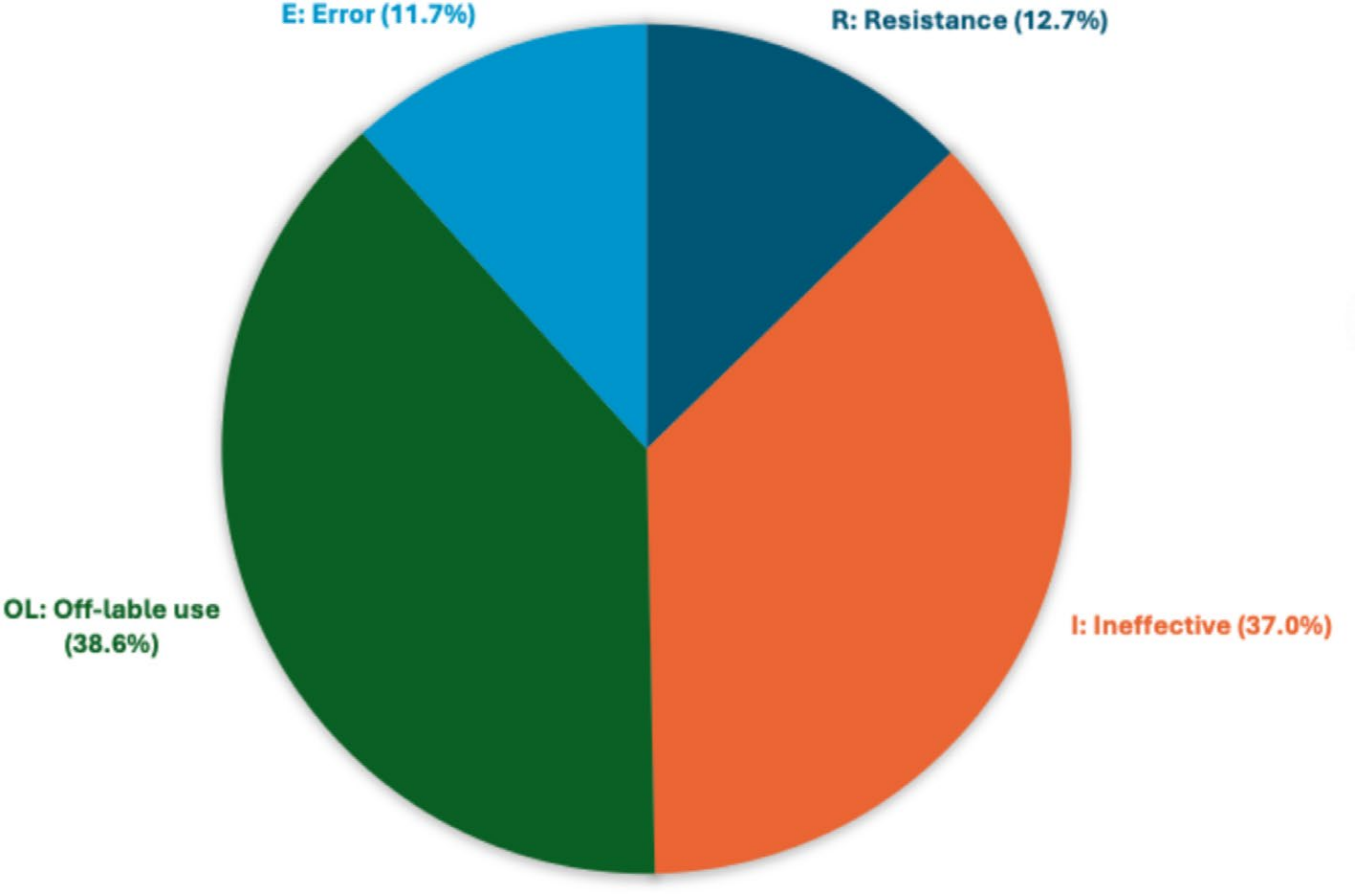
Distribution of reports by RIOLE categories (*n* = 68 MedDRA Preferred Terms, *n* = 2958 drug‐AE pairs). Drug‐AE pair: Since each ICSR may include multiple drugs and multiple reported events, the drug–event pair is where each pair represents one suspected/interacted drug reported with one adverse event within an ICSR. RIOLE: The PT of each adverse event has been grouped by RIOLE categories, where PTs that suggest “resistance,” “ineffectiveness,” “ off‐label use,” or “errors.”

**FIGURE 5:**
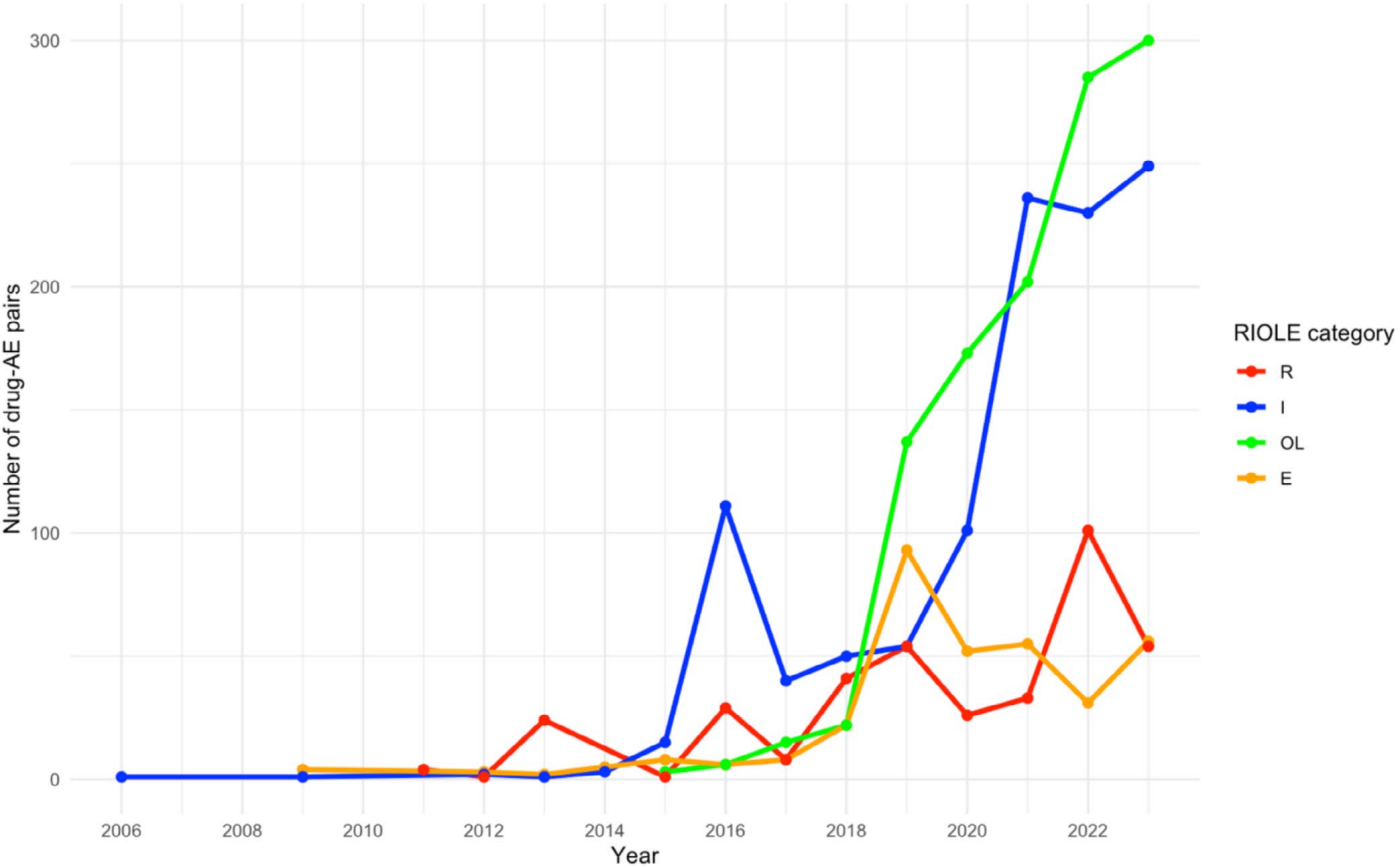
Distribution of reports by RIOLE categories over time (*n* = 2958 drug‐AE pairs). Drug‐AE pair: Since each ICSR may include multiple drugs and multiple reported events, the drug–event pair is where each pair represents one suspected/interacted drug reported with one adverse event within an ICSR. RIOLE: The PT of each adverse event has been grouped by RIOLE categories, where PTs that suggest “resistance,” “ineffectiveness,” “off‐label use,” or medication “errors.”

The PT of each adverse event have been grouped by RIOLE categories, where PTs that suggests “off‐label use” category represented the largest category, accounting for (1143; 38.6%) drug‐AE pairs, followed by “ineffectiveness” category with (1094; 37.0%). PT terms categorized as “resistance” category comprised (376; 12.7%), while medication “errors” accounted for (345; 11.7%). Collectively, “ off‐label use” and “ineffectiveness” categories together constituted over 70% of all extracted drug‐AE pairs, (Figure 4). The reporting of PTs of “off‐label use” and “ineffectiveness” categories grew sharply after 2017, while “resistance” and “errors” stayed much lower and showed only small ups and downs across the years (Figure 5).

Within the “resistance” category nine PTs were identified; the most frequently reported PTs were *Drug resistance* (210; 7.0%) and *Pathogen resistance* (132; 4.5%). Other less frequently reported PTs included *Multiple‐drug resistance* (10; 0.3%). The antibiotics most often linked to “resistance” category were meropenem, levofloxacin, streptomycin, ofloxacin, and amikacin. The “ineffectiveness” group (1094; 37.0%) includes 19 PTs; the most reported PT was *Drug ineffective* (751; 25.4%), followed by *Treatment failure* (131; 4.4%), *Therapy nonresponder* (40; 1.4%), and *Therapeutic response decreased* (36; 1.2%). These reports were mainly for clofazimine, pyrazinamide, meropenem, azithromycin, and ceftriaxone. The “off‐label use” group (1143; 38.6%) contains four PTs, and the PT *Off‐label use* accounted for (795; 26.4%), and it was the most reported term among all four categories. Lastly, the “error” category (345; 11.7%) comprised 36 PTs, with the most frequently reported PT being *Product use issue* (60; 2.0%) followed by *Medication error* (44; 1.5%) (Table 3).

**TABLE 3:** Distribution of MedDRA preferred terms and the top five reported antibiotics within each RIOLE category.

Top reported preferred term by RIOLE categories		Top 5 reported antibiotics per RIOLE categories	
R: “resistance,” I: “ineffective,” OL: “off‐label use,” E: “error”	Drug‐AE pairs (total 2958) (*n*, %)	Antibiotics	Drug‐AE pairs (total 2958) (*n*, %)
R			
Preferred term (PT), total = 9 PTs^a^	**376 (12.7%)**		
Drug resistance	210 (7.0%)	Meropenem	39 (1.32%)
Pathogen resistance	132 (4.5%)	Levofloxacin	32 (1.8%)
Bacterial infection	10 (0.3%)	Streptomycin	32 (1.8%)
Multiple‐drug resistance	10 (0.3%)	Ofloxacin	31 (1.05%)
		Amikacin	29 (01.0%)
I			
Preferred term (PT), total = 19 PTs^a^	**1094 (37.0%)**		
Drug ineffective	751 (25.4%)	Clofazimine	165 (5.6%)
Treatment failure	131 (4.4%)	Pyrazinamide	136 (4.6%)
Therapy nonresponder	40 (1.4%)	Meropenem	112 (3.8%)
Therapeutic response decreased	36 (1.2%)	Azithromycin	81 (2.7%)
Drug intolerance	27 (0.9%)	Ceftriaxone	73 (2.5%)
Paradoxical drug reaction	26 (0.8%)		
Therapeutic product effect delayed	21 (0.7%)		
Disease progression	14 (0.5%)		
Therapeutic product effect incomplete	14 (0.5%)		
Therapeutic response unexpected	11 (0.4%)		
OL			
Preferred term (PT), total = 4 PTs^a^	**1143 (38.6%)**		
Off label use	795 (26.9%)	Azithromycin	305 (10.3%)
Product use in unapproved indication	269 (9.1%)	Ceftriaxone	254 (8.6%)
Drug ineffective for unapproved indication	77 (2.6%)	Meropenem	117 (4.0%)
		Clofazimine	69 (2.3%)
		Linezolid	69 (2.3%)
E			
Preferred term (PT), total = 36 PTs^a^	**345 (11.7%)**		
Product use issue	60 (2.0%)	Amoxicillin; Clavulanic acid	81 (2.7%)
Medication error	44 (1.5%)	Linezolid	43 (1.5%)
Incorrect dose administered	37 (1.3%)	Clofazimine	36 (1.2%)
Intentional product use issue	35 (1.2%)	Metronidazole	30 (1.0%)
Intentional product misuse	34 (1.1%)	Ceftriaxone	25 (0.9%)
Product prescribing error	26 (0.9%)		
Overdose	16 (0.5%)		
Treatment noncompliance	14 (0.4%)		

*Note:* Bold valuse are the total of each category.

^a^ PTs reported fewer than 10 times are not shown in this table; the full PT list is available in Table S1.

As shown in Table 4, all examined variables were significantly associated with RIOLE categories. After excluding unknown values, gender showed a significant association with RIOLE [ *χ* ^2^(3) = 74.3, *p* < 0.001], although the effect was small (Cramer's V = 0.17), with males representing a larger percentage (60%) of ineffectiveness (I) reports, while females contributed proportionally more (52%) to error (E) reports. Reporter type also differed across RIOLE categories (*χ* ^2^ = 127.42, *p* = 0.0001). Drug ATC class was also with significant association to RIOLE, with a small‐to‐moderate association between RIOLE and ATC categories (Pearson's *χ* ^2^ = 167.49, df = 3, *p* < 0.001; Cramer's V = 0.24), with J04 antimycobacterials contributing a higher proportion of ineffectiveness cases (27.2%) compared with the other categories.

**TABLE 4:** Association between RIOLE categories and selected variables.

	RIOLE				
	R	I	OL	E	*χ* ^2^, *p*
	376 (100%) (*n*, %)	1094 (100%) (*n*, %)	1143 (100%) (*n*, %)	345 (100%) (*n*, %)	
Sex					*χ* ^2^ = 74.3
Male	220 (58.5%)	658 (60.1%)	477 (41.7%)	147 (42.6%)	*p* < 0.001
Female	111 (29.5%)	330 (30.2%)	432 (29.5%)	181 (52.5%)	
Reporter					*χ* ^2^ = 127.42
Healthcare professional	373 (99.2)	1051 (96.1%)	1108 (96.9%)	290 (84.1%)	*p* = 0.0001*
Nonhealthcare professional	3 (0.8%)	40 (3.7%)	33 (2.9%)	55 (15.9%)	
AWaRe					*χ* ^2^² = 421.8
Access	77 (20.5%)	224 (20.5%)	120 (10.5%)	134 (38.8%)	*p* < 0.001
Watch	249 (66.2%)	517 (47.3%)	847 (74.1%)	126 (36.5%)	
Reserve	14 (3.7%)	32 (2.9%)	69 (6%)	43 (12.5%)	
Others	36 (9.6%)	321 (29.3%)	107 (9.4%)	42 (12.2%)	
Drug ATC classification					*χ* ^2^ = 167.49
J01	341 (90.7%)	796 (72.8%)	1046 (91.5%)	303 (87.8%)	*p* < 0.001
J04	35 (9.3%)	298 (27.2%)	97 (8.5%)	42 (12.2%)	
Reaction outcome					*χ* ^2^ = 99.7
Fatal	19 (5.1%)	157 (14.4%)	26 (2.3%)	9 (2.6%)	*p* = 0.0001*
Not recovered/not resolved	53 (14.1%)	48 (4.4%)	2 (0.2%)	7 (2%)	
Recovered/recovering^a^	72 (19.2)	106 (9.7%)	33 (2.9%)	41 (11.9%)	
WHO region					*χ* ^2^ = 191.31
African Region	3 (0.8%)	15 (1.4%)	30 (2.6%)	4 (1.2%)	*p* = 0.0001*
Eastern Mediterranean Region	61 (16.2%)	165 (15.1%)	773 (27.6%)	160 (46.4%)	
South–East Asia Region	312 (83%)	875 (80%)	773 (67.6%)	173 (50.1%)	
Western Pacific Region	0 (0%)	39 (3.6%)	24 (2.1%)	8 (2.3%)	

*Note:* *p‐values were computed using chi‐squared test with Monte Carlo simulation (10 000 replicates) due to small frequencies [28]. Totals may not sum to 100% because “Unknown” categories were excluded from the chi‐squared analyzes. R: “resistance,” I: “ineffective,” OL: “off‐label use,” E: “error.”

^a^ Recovered/resolved, recovered/resolved with sequelae, and recovering/resolving were combined together.

Reaction outcome varied across RIOLE categories, and this association persisted even after excluding unknown outcomes (*χ* ^2^ = 99.7, *p* = 0.0001), with a moderate effect size (Cramer's V = 0.29), with fatal (157;14.4%) and not‐recovered outcomes (53;14.1%) more frequently observed among ineffectiveness and resistance reports, respectively. Antibiotic AWaRe categories were also unevenly distributed across RIOLE groups, showing a statistically significant and small‐to‐moderate association (Pearson's *χ* ^2^ = 421.8, df = 9, *p* < 0.001; Cramer's V = 0.22), Watch antibiotics predominated in both the ineffectiveness (517; 47.3%) and off‐label use (847; 74.1%) groups, whereas Access antibiotics accounted for a larger proportion of error‐related reports (38.8%).

The WHO region demonstrated a significant association with RIOLE (Pearson's *χ* ^2^ with Monte Carlo simulation, *χ* ^2^ = 191.31, *p* = 0.0001), although the effect was small (Cramer's V = 0.15). Reporter type also differed significantly, with a small‐to‐moderate association after removing unknown values (Pearson's *χ* ^2^ with Monte Carlo simulation *χ* ^2^ = 127.42, *p* = 0.0001; Cramer's V = 0.21), with healthcare professional reports forming a relatively higher proportion (1108; 96.9%) of OL cases compared with the other groups.

Out of the total 2958 drug‐AE pairs, the majority were from the *Watch* group (*n* = 1739; 58.8%), followed by *Access* (*n* = 555; 18.8%), Others (*n* = 506; 17.1%), and *Reserve* (*n* = 158; 5.3%). Among *Access* the amoxicillin/clavulanic acid (198; 6.7%), amikacin (98; 3.3%), and metronidazole (84; 2.8%) were the most frequently reported. The main reaction terms were *Drug ineffective* (166; 5.6%), *Off‐label use* (67; 2.3%), and *Pathogen resistance* (50; 1.7%). Within the *Watch* group, the highest involved azithromycin (406; 13.7%), ceftriaxone (380; 12.8%), and meropenem (289; 9.8%) with the most common PTs were *Off‐label use* (628; 21.2%), *Drug ineffective* (371; 12.5%), and *Product use in unapproved indication* (165; 5.6%). For the *Reserve* category, linezolid accounted for all reports (158; 5.3%), with *Off‐label use* (42; 1.4%) and *Product use in unapproved indication* (26; 0.9%) being the most frequent PTs, (Table 5).

**TABLE 5:** Distribution of antibiotics according to AWaRe classification system and the top five reported preferred terms (PTs) within each AWaRe category.

AWaRe		Top 5 reported Preferred terms (PTs) per AWaRe Categories		
Antibiotics per AWaRe	Drug‐AE pairs (total 2958) (*n*, %)	PT	Drug‐AE pairs (total 2958) (*n*, %)	RIOLE
Access				
Amoxicillin; clavulanic acid	198 (6.7%)	Drug ineffective	166 (5.6%)	I
Amikacin	98 (3.3%)	Off label use	67 (2.3%)	OL
Metronidazole	84 (2.8%)	Pathogen resistance	50 (1.7%)	R
Clindamycin	45 (1.5%)	Product use in unapproved indication	44 (1.5%)	OL
Gentamicin	42 (1.4%)	Drug resistance	25 (0.8%)	R
Ampicillin	31 (1.0%)			
Sulfamethoxazole; trimethoprim	28 (0.9%)			
Amoxicillin	25 (0.8%)			
Tetracycline	2 (0.09%)			
Trimethoprim	2 (0.09%)			
Total	555 (18.8%)			
Watch				
Azithromycin	405 (13.7%)	Off label use	628 (21.2%)	OL
Ceftriaxone	380 (12.8%)	Drug ineffective	371 (12.5%)	I
Meropenem	289 (9.8%)	Product use in unapproved indication	165 (5.6%)	OL
Levofloxacin	141 (4.8%)	Drug resistance	146 (4.9%)	R
Moxifloxacin	113 (3.8%)	Pathogen resistance	80 (2.7%)	R
Vancomycin	109 (3.7%)			
Imipenem	74 (2.5%)			
Ofloxacin	62 (2.1%)			
Streptomycin	55 (1.9%)			
Piperacillin; tazobactam	38 (1.3%)			
Cefotaxime	28 (0.9%)			
Clarithromycin	25 (0.8%)			
Ciprofloxacin	14 (0.5%)			
Gatifloxacin	3 (0.1%)			
Minocycline	2 (0.09%)			
Moxifloxacin; Tobramycin	1 (0.01%)			
Total	1739 (58.8%)			
Reserve				
Linezolid	158 (5.3%)	Off label use	42 (1.4%)	OL
Total	158 (5.3%)	Product use in unapproved indication	26 (0.9%)	OL
		Drug ineffective	19 (0.6%)	I
		Intentional product use issue	12 (0.4%)	E
		Product use issue	12 (0.4%)	E
Others		Drug ineffective	195 (6.6%)	I
Clofazimine^a^	278 (9.4%)	Treatment failure	80 (2.7%)	I
Pyrazinamide^b^	199 (6.7%)	Off label use	58 (2.0%)	OL
Minocycline^c^	29 (1.0%)	Product use in unapproved indication	34 (1.1%)	OL
Total	506 (17.1%)	Drug resistance	30 (1.0%)	R

^a^ Not categorized.

^b^ Not categorized as a standard *Access*, *Watch*, or *Reserve* antibiotic because it is a first‐line antituberculosis drug.

^c^ Depends on the route of administration; if oral classified as *Watch*, if injection classified as reserve.

## 4 Discussion

### 4.1 Principal Findings

This study analyzed antibacterial‐related reports from LMICs that are members of the WHO PIDM and submitting reports to VigiBase. No reports were retrieved from LMICs in the WHO region of Americas, or from LMICs located in Europe and Oceania continents. Although VigiBase has collected spontaneous adverse reaction reports since 1968, the first eligible antibiotic‐related report meeting the study criteria was submitted in 2006. A notable increase in the number of reports was observed after 2016, reflecting a steady growth in PV activity in LMICs related to antibiotic resistance and inappropriate use. The marked increase in reports after 2016 may reflect improvements in national pharmacovigilance infrastructure (e.g., better electronic reporting systems, training, policy emphasis) and heightened PV awareness more than a sudden jump in antibiotic failure. PV systems in LMICs have been gradually strengthened over the past decade, enabling more frequent detection and submission of ADRs, including those with resistance and inappropriate use related terms [29].

The dominance of reports from the South‐East Asia and Eastern Mediterranean regions may reflect several regional characteristics. First, both regions have some of the highest infectious disease burdens globally, particularly tuberculosis, which leads to extensive use of J04 antimycobacterial agents. Second, studies show that these regions also have higher overall antibiotic consumption, including frequent use of broad‐spectrum agents and higher rates of inappropriate prescribing [30, 31, 32, 33]. Given the population size of countries such as India and Egypt, both of which also have more established PV reporting systems compared with other LMICs, their contribution likely drives part of the higher volume of reports observed in VigiBase. According to the WHO Global Benchmarking Tool as of October 2025, India and Egypt have achieved regulatory Maturity Level 3 (ML3), indicating functional regulatory governance and structured national PV systems capable of supporting more systematic adverse event reporting [34]. Although the African region includes four LMICs with ML3 status, the rest of LMICs included in the analysis remain at earlier stages of regulatory development [34], which may contribute to the uneven reporting patterns observed.

The top two reported PTs across all RIOLE were *Off‐label use* and *Drug ineffective*, accounted for approximately one quarter each of all drug‐AE pairs (795; 26.9% and 25.4%, respectively), far exceeding explicit resistance terms such as *Drug resistance* (210; 7.0%), *Pathogen resistance* (132; 4.5%), and *Multiple‐drug resistance* (10; 0.3%). These distributions differ from the pattern observed in EudraVigilance database (the European PV database) analysis of ceftazidime and avibactam ADR reports, where *Drug resistance* was the most frequently reported PT (ICSRs = 69; 53.5%) followed by *Drug ineffective* (ICSRs = 54; 41.9%) [10]. Another finding from analysis of reports of selected antimicrobials from VigiBase via VigiAccess (a free and public web tool that provides access to VigiBase) showed that the most reported three terms were *Drug ineffective*, *Pathogen resistance*, and *Off‐label use* (2707; 46.7%, 749; 13.0%, and 533; 9.0%, respectively) [6].

The prominence of *Off‐label use* as the most frequently reported PT, additionally across RIOLE categories, “off‐label use” (38.6%) and “ineffectiveness” (37.0%) categories together represented over two‐thirds of all reports suggesting several structural and clinical realities in LMICs health systems. Off‐label prescribing of antibiotics is common in settings where diagnostic confirmation is limited [35], we did not retrieve reports for PT *Antimicrobial susceptibility test resistant* and *Antimicrobial susceptibility test intermediate* (Table S1) while retrieved only for the PT *Antimicrobial susceptibility test sensitive* (34; 1.13%). Moreover, in LMICs treatment pathways are guided by empirical decision‐making, and national guidelines may be inconsistently implemented. In such contexts, clinicians often prescribe outside approved indications to expand treatment options when microbiological evidence is unavailable. The high proportion of ineffectiveness reports may also reflect poor‐quality or substandard antibiotics, interrupted treatment courses, delayed care‐seeking, variable drug quality, and supply‐chain issues (e.g., substandard formulations) common in LMIC settings [36], all of which increase the likelihood of treatment failure and resistance [9].

Although reports of PTs in “resistance” category were reported less frequently (12.5%), their presence still indicates that suspected emerging resistance is being captured by PV systems despite the known under‐reporting issue. It is important to emphasize that these reports represent suspicions by the reporter, not confirmed resistance, as most cases lack objective microbiological confirmation. Nevertheless, this is precisely where the added value of PV lies for AMR surveillance: in settings where access to specialized microbiology laboratories is limited or unavailable, the ability of healthcare professionals to communicate a clinical suspicion of resistance or treatment failure through PV reporting provides valuable real‐world data insight. Findings from an in‐depth analysis of antibiotics ICSRs from VigiBase confirmed that PTs linked to resistance (e.g., *Pathogen resistance, Drug resistance*) have high positive predictive value, with over 90% of “probable” reports representing true antibiotic resistance when narratives were reviewed [11]. PV centers in LMICs should therefore actively promote the use of these resistance‐ and ineffectiveness‐related MedDRA terms among reporters, as a practical means to increase the volume and quality of AMR‐relevant reports and contribute to early detection of suspected resistance where conventional laboratory‐based surveillance is absent.

Although errors‐related PTs were the lowest reported (11.7%), the PTs of *Medication error*, *Incorrect dose administered*, *Product use issue*, and *Treatment noncompliance* highlight important weaknesses in the medication‐use process. These PTs point to challenges such as dosing inaccuracies, administration mistakes, and inconsistent patient adherence, all of which are well‐recognized contributors to suboptimal antibiotic exposure and potential treatment failure. The presence of these error‐related PTs in the findings suggests that system‐level factors, including limited prescribing support, inconsistent treatment monitoring, and patient‐level barriers, may be influencing antibiotic outcomes in LMICs settings.

Within the AWaRe classification, *Watch* antibiotics accounted for the majority of drug–AE pairs (1739; 58.8%), of which 847 drug–AE within the OL category, where azithromycin, ceftriaxone, and meropenem was predominantly reported as suspect antibiotics, suggesting that inappropriate prescribing of broad‐spectrum agents is a recognized and reportable concern. Results from a prevalence survey in 69 countries showed that *Watch* antibiotic use is disproportionately high in LMICs hospitals; this reflects increased availability of broad‐spectrum agents, rising AMR pressures, and higher infectious disease burden [37]. Global sales data also show that from 2000 to 2015, *Watch* consumption rose faster than *Access* antibiotics, especially in LMICs [37, 38]. Moreover, the predominance of PTs of *Off‐label use* and *Drug ineffective* among *Watch* antibiotics further points to the challenges clinicians face when managing severe infections with limited diagnostic support, leading to reliance on empirical escalation, off‐guideline prescribing, and trial‐and‐error treatment patterns [37, 39]. *Watch* antibiotics, especially broad‐spectrum antibiotics, are more available and more frequently dispensed (often without prescription) than *Access* agents, partly because markets are saturated with branded generics of third‐generation cephalosporins and fluoroquinolones [40].

Access antibiotics were primarily associated with PTs reflecting inappropriate indication and therapeutic failure PTs of *Drug ineffective, Off‐label use, Product use in unapproved indication*, consistent with their wide availability and frequent use. *Reserve* antibiotics, primarily linezolid, were disproportionately associated with off‐label use and ineffectiveness; this may reflect that *Reserve* antibiotics are usually last line drugs used in more complicated cases where failure or off‐label use is more likely to draw attention [39]. These findings highlight important stewardship gaps, demonstrating that inappropriate selection, empirical escalation to *Watch* antibiotics, and off‐label use of *Access* and *Reserve* antibiotics are contributors to safety concerns that can be captured in LMICs PV data.

The four RIOLE categories could be grouped into two broader categories to differentiate between reports suggesting that the antibiotic did not provide the expected therapeutic benefit (R and I categories) and reports indicating that the antibiotic was not used appropriately (OL and E categories). Combining “resistance” and “ineffectiveness” (RI) yielded 1470 drug–AE pairs (49.7%), while “off‐label use” and “errors” (OLE) accounted for 1488 pairs (50.3%), Figure 4. Within the RI group, fatal outcomes (19; 5.1% and 157; 14.4%, respectively) and not‐recovered outcomes (53; 14.1% and 48; 4.4%, respectively) were observed, Table 4. Furthermore, J04 antimycobacterial contributed (27.2% of ineffectiveness cases and 9.3% of resistance cases), reflecting the challenge of tuberculosis treatment failure in LMICs. By contrast, the OLE group was dominated by *Watch* antibiotics (847; 74.1% of off‐label use reports and 126; 36.5% of error of use). The grouping of RIOLE to RI and OLE reports helps to capture fundamentally different problems: reported RI related terms require investigation into suspected treatment failure and suspected resistance, whereas reported OLE related terms point to prescribing, dispensing, or administration practices that are potentially preventable through guideline implementation, prescriber, healthcare professionals, and patients education, and regulatory enforcement.

In this study we extracted additional PTs from MedDRA dictionary, beyond those identified in the literature [6, 7, 9, 10], and mapped them to the RIOLE framework [6]. Many antibiotic‐related ICSRs reflect complex clinical trajectories that cannot be reliably interpreted from PTs alone. For example, terms such as *Bacterial sepsis* or *Bacteroides bacteraemia* may represent true antimicrobial resistance when a pathogen fails to respond to appropriate therapy, but the same PTs may indicate ineffectiveness due to suboptimal dosing, inappropriate spectrum selection or delayed initiation. Likewise, PTs such as *Coinfection, Therapeutic incompatibility*, and *Therapy cessation* may reflect issues including new coinfecting by an intrinsically resistant pathogen, drug–drug interactions, nonadherence, or preventable errors, depending on the additional narrative available. The national pharmacovigilance centers, with access to full narratives and the ability to request targeted follow‐up information, are positioned to differentiate between inappropriate antibiotic choice, dosing errors, intolerance leading to treatment interruption, and true pathogen resistance [11]. Leveraging these richer data sources is therefore essential to correctly interpret these clinically heterogeneous signals and support more effective antimicrobial stewardship.

### 4.2 Strengths and Limitations

A key strength of this study lies in being the first LMICs‐wide analysis to apply the RIOLE related MedDRA PTs to PV data and integrate them with the WHO AWaRe classification, enabling regional comparison. It also provides an extensive list of MedDRA terms that establish a foundation for future reproducibility or integration into routine PV monitoring. The study generates baseline evidence supporting the feasibility of integrating PV data into the AMR surveillance, underscoring the need for complementary, data‐driven monitoring systems in resource‐limited settings.

This study has some limitations. First, the analysis reflects only reports submitted to VigiBase from LMICs; reporting is disproportionately driven by a few high‐reporting countries, while LMICs contribute comparatively few ICSRs [34]; therefore, the findings may not fully represent the underlying burden of antibiotic‐related problems in these settings. Second, spontaneous reporting is inherently subject to under‐reporting, and the likelihood of submitting a report depends heavily on clinical suspicion, reporter awareness, and local PV culture. As a result, the true frequency of resistance, ineffectiveness, or misuse events is likely higher than captured in these data. Third, some variables contained a substantial proportion of unknown entries, particularly for reaction outcomes and reporter type. Although these values were retained in descriptive summaries, they were excluded from inferential analyzes, which may have introduced bias and reduced the precision of the observed associations. Fourth, we did not review the case narratives, which might or might not include confirmatory clinical information such as microbiological results, treatment context, or detailed dosing regimens, making it difficult to establish causal relationships between the reported event and the suspected antibiotic. Finally, variability in how healthcare professionals recognize, document, and code antibiotic‐related problems, particularly distinguishing between resistance, therapeutic failure, and inappropriate use, may introduce subjective selection of the appropriate MedDRA term. Strengthening training for healthcare professionals on how to report and code antibiotic treatment failures, including suspected lack of efficacy, resistance, and use‐related issues [41], would improve the accuracy and utility of PV data for antimicrobial stewardship and AMR surveillance in LMIC settings.

## 5 Clinical and Policy Implications

The patterns observed across RIOLE categories and AWaRe groups highlight important implications for both clinical practice and policy in LMICs. The predominance of *Drug ineffective* and *Off‐label use* PTs, particularly among *Watch* antibiotics, suggests heavy reliance on empirical treatment prescribing, which contributes to therapeutic failure and may accelerate resistance development. This underscores the need for policy measures that strengthen guideline‐based prescribing and expand access to clinical decision‐support tools at the point of care to reduce trial‐and‐error prescribing and prevent avoidable treatment failures.

The findings also emphasize the importance of stronger national PV systems with the capacity to review the ICSRs narratives and request follow‐up information, as these local insights are essential for accurately distinguishing resistance, ineffectiveness, and misuse. Integrating local PV centers into national AMR policies could substantially improve early detection of inappropriate antibiotic use and emerging resistance while guiding targeted stewardship interventions. Although such approaches have been implemented in India [42], similar integration is needed across LMICs.

As LMICs continue to advance their PV and regulatory maturity, leveraging ICSRs, especially when combined with narrative review and laboratory data, has the potential to fill critical surveillance gaps and improve clinical outcomes in infectious disease management. Policy efforts should, therefore, prioritize improving reporting and coding of antibiotic treatment failures, investing in clinician training, and strengthening stewardship oversight of *Watch* antibiotics.

## 6 Research Implications

The findings of this study highlight several directions for future research. First, there is a need to define what constitutes an ideal AMR case report in PV, as developing such a standard would greatly enhance the interpretability of ICSRs for AMR monitoring. Second, the PTs identified as potential indicators of resistance or misuse require formal validation studies to determine their accuracy, specificity, and predictive value in LMICs settings. Mixed‐methods research combining PV narratives, chart review, and diagnostic data is also needed to understand why antibiotic therapy fails; this includes linking PTs patterns with clinical records, microbiology results, and stewardship audits. Finally, the AWaRe–RIOLE associations observed in this study underscore the need for implementation research focused on strengthening ADR reporting quality in LMICs, including interventions that train healthcare professionals to actively motivate and encourage their engagement in recognizing and coding antibiotic treatment failures, integrating PV with AMR surveillance. Together, these efforts would refine the RIOLE framework, strengthen the utility of ICSRs for AMR signal detection, and support more targeted stewardship interventions.

## 7 Conclusion

This study demonstrates that antibiotic‐related ICSRs from LMICs contain reports that might suggest suspected resistance and inappropriate use. The prominence of ineffectiveness and off‐label use underscores the challenges clinicians face in settings with limited diagnostic support. These findings also highlight the added value of PV systems as a complementary surveillance mechanism for antimicrobial resistance, especially where microbiology capacity is limited. AMR‐related data scarcity in LMICs is well recognized, and the WHO PV network provides an existing infrastructure capable of capturing suspected resistance signals and inappropriate use through routinely collected ICSRs.

Furthermore, the four RIOLE categories can be further consolidated into two actionable dimensions for stewardship purposes: suspected resistance and ineffectiveness (RI), indicating that the antibiotic may not have provided the expected therapeutic benefit, and off‐label use and errors (OLE), indicating that the antibiotic was not used appropriately. This regrouping allows policymakers and PV centers to tailor their interventions accordingly. Overall, strengthening national PV capacity, improving reporting quality, and integrating PV insights with stewardship and AMR surveillance efforts could enhance early detection of emerging resistance patterns and support more rational antibiotic use across LMICs health systems.

## Author Contributions

H.S., C.S.L., and M.S. contributed to the study conception. H.S. established the search strategy. H.S. and J.M. contributed to data acquisition from UMC. H.S. analyzed the data and wrote the first draft of the manuscript. All authors contributed to the interpretation of the results and to manuscript revision.

## Funding

Karolinska Institute travel grant, project number KI99519253 and Research Council of Norway, project number 103287101.

## Conflicts of Interest

The authors declare no conflicts of interest.

## Supporting information


**Table S1:** MedDRA preferred terms relevant to antimicrobial resistance, therapeutic ineffectiveness, off‐label use, and errors of use identified and used for data extraction and analysis from VigiBase.

## Data Availability

Research data are not shared.
