# Supplementary material for: The Role of Pharmacovigilance Database in Identifying Antibiotic Resistance and Inappropriate Use: An Analysis of VigiBase Reports From Lower‐Middle‐Income Countries
Source: Pharmacoepidemiol Drug Saf. 2026 Jun 8;35(6):e70404. doi: 10.1002/pds.70404 (PMC13247192; doi:10.1002/pds.70404)
Supplement: Supplementary file 1 — Table S1: MedDRA preferred terms relevant to antimicrobial resistance, therapeutic ineffectiveness, off‐label use, and errors of use identified and used for data extraction and analysis from VigiBase. [file PDS-35-e70404-s001.docx]

**Supplementary Table 1:** MedDRA^®^ preferred terms relevant to antimicrobial resistance, therapeutic ineffectiveness, off-label use identified and used for data extraction from VigiBase.

| **System Organ Classes** (**SOCs)** |  | **Preferred Term (PT)** | **MedDRA**^®^ **Code** | **RIOLE Category** | Source |
| --- | --- | --- | --- | --- | --- |
| Infections and infestations | 1 | Atypical mycobacterial infection | 10061663 | R | Co-reported |
|  | 2 | Bacterial infection | 10060945 | R | Co-reported |
|  | 3 | Bacterial sepsis | 10053840 | R | Co-reported |
|  | 4 | Bacteroides bacteraemia | 10058853 | R | Co-reported |
|  | 5 | Coinfection | 10083750 | R | Co-reported |
|  | 6 | Infection reactivation | 10070891 | I | Co-reported |
|  | 7 | Pathogen resistance | 10034133 | R | Literature |
| General disorders and administration site conditions | 8 | Absence of immediate treatment response^*^ | 10081766 | I | Extracted^#^ |
|  | 9 | Atypical dose response relationship^*^ | 10081873 | I | Extracted |
|  | 10 | Decreased activity | 10011953 | I | Literature |
|  | 11 | Disease progression | 10061818 | I | Co-reported |
|  | 12 | Disease recurrence | 10061819 | I | Co-reported |
|  | 13 | Drug effect less than expected^*^ | 10083365 | I | Extracted |
|  | 14 | Drug ineffective | 10013709 | I | Literature |
|  | 15 | Drug ineffective for unapproved indication | 10051118 | I and OL | Literature |
|  | 16 | Drug intolerance | 10061822 | I | Extracted |
|  | 17 | Drug Resistance | 10059866 | R | Literature |
|  | 18 | Drug therapeutic incompatibility^*^ | 10053581 | I | Extracted |
|  | 19 | Drug tolerance^*^ | 10052804 | I | Extracted |
|  | 20 | Drug tolerance increased^*^ | 10052806 | R | Extracted |
|  | 21 | Drug tolerance decreased | 10052805 | I | Co-reported |
|  | 22 | Loss of therapeutic response | 10084221 | I | Extracted |
|  | 23 | Missing dose response relationship^*^ | 10081872 | E | Extracted |
|  | 24 | Multiple drug resistance | 10048723 | R | Literature |
|  | 25 | Paradoxical drug reaction | 10048958 | I | Extracted |
|  | 26 | Remission not achieved^*^ | 10076313 | I | Extracted |
|  | 27 | Therapeutic product effect decreased | 10082201 | I | Extracted |
|  | 28 | Therapeutic product effect delayed | 10082202 | I | Extracted |
|  | 29 | Therapeutic product effect incomplete | 10082200 | I | Extracted |
|  | 30 | Therapeutic product effect variable^*^ | 10082204 | I | Extracted |
|  | 31 | Therapeutic product ineffective | 10060769 | I | Literature |
|  | 32 | Therapeutic product ineffective for unapproved indication^*^ | 10060770 | I and LO | Extracted |
|  | 33 | Therapeutic response decreased | 10043414 | I | Literature |
|  | 34 | Therapeutic response delayed | 10053181 | I | Extracted |
|  | 35 | Therapeutic response unexpected | 10043417 | I | Literature |
|  | 36 | Therapy non-responder | 10051082 | I | Extracted |
|  | 37 | Therapy partial responder | 10078115 | I | Extracted |
|  | 38 | Treatment Failure | 10066901 | I | Literature |
| Injury, poisoning, and procedural complications | 39 | Accidental overdose | 10000381 | E | Extracted |
|  | 40 | Accidental underdose | 10074904 | E | Extracted |
|  | 41 | Contraindicated product administered | 10076476 | E and OL | Literature |
|  | 42 | Contraindicated product prescribed | 10081744 | E | Co-reported |
|  | 43 | Inappropriate schedule of product administration | 10081572 | E | Co-reported |
|  | 44 | Incorrect dosage administered^*^ | 10073768 | E | Extracted |
|  | 45 | Incorrect dose administered | 10064355 | E | Extracted |
|  | 46 | Incorrect product administration duration | 10081581 | E | Co-reported |
|  | 47 | Intentional overdose | 10022523 | E | Extracted |
|  | 48 | Intentional product misuse | 10074903 | E | Extracted |
|  | 49 | Intentional product misuse to child^*^ | 10084403 | E | Extracted |
|  | 50 | Intentional product use issue | 10076308 | E | Co-reported |
|  | 51 | Intercepted product prescribing error | 10081743 | E | Co-reported |
|  | 52 | Medication error | 10027091 | E | Literature |
|  | 53 | Off label use | 10053762 | OL | Literature |
|  | 54 | Overdose | 10033295 | E | Extracted |
|  | 55 | Prescribed overdose | 10051076 | E | Extracted |
|  | 56 | Prescribed underdose | 10073085 | E | Co-reported |
|  | 57 | Prescription drug used without a prescription | 10076639 | E | Co-reported |
|  | 58 | Product administered to patient of inappropriate age | 10081578 | E | Co-reported |
|  | 59 | Product administration error | 10081576 | E | Co-reported |
|  | 60 | Product communication issue | 10080099 | E | Co-reported |
|  | 61 | Product dose omission issue | 10084406 | E | Co-reported |
|  | 62 | Product preparation issue | 10081301 | E | Co-reported |
|  | 63 | Product prescribing error | 10081770 | E | Literature |
|  | 64 | Product prescribing issue | 10080459 | E | Co-reported |
|  | 65 | Product selection error | 10076542 | E | Co-reported |
|  | 66 | Product storage error | 10079843 | E | Co-reported |
|  | 67 | Product use in unapproved indication | 10076476 | OL | Literature |
|  | 68 | Product use issue | 10076309 | E | Co-reported |
|  | 69 | Therapeutic product effective for unapproved indication^*^ | 10079316 | OL | Extracted |
|  | 70 | Treatment noncompliance | 10049414 | E | Extracted |
|  | 71 | Underdose | 10057362 | E | Co-reported |
|  | 72 | Wrong drug | 10080306 | E | Co-reported |
|  | 73 | Wrong dose | 10080304 | E | Extracted |
|  | 74 | Wrong technique in product usage process | 10076573 | E | Co-reported |
| Investigations | 75 | Antimicrobial susceptibility test resistant^*^ | 10002793 | R | Extracted |
|  | 76 | Antimicrobial susceptibility test intermediate^*^ | 10002792 | R | Extracted |
|  | 77 | Bacterial infection index increased^*^ | 10085801 | R | Extracted |
|  | 78 | Bacterial test positive | 10059421 | R | Co-reported |
| Psychiatric disorders | 79 | Drug abuse | 10013654 | E | Co-reported |
| Product issues | 80 | Product complaint | 10069902 | E | Co-reported |
|  | 81 | Product quality issue | 10069327 | E | Co-reported |
|  | 82 | Suspected counterfeit product | 10071287 | E | Co-reported |
| Surgical and medical procedures | 83 | Product used for unknown indication | 10070592 | OL and E | Co-reported |
|  | 84 | Self-medication | 10050729 | E | Co-reported |
|  | 85 | Therapy cessation | 10065154 | E and I | Co-reported |

R: Resistance, I: Ineffective, OL: Off-label use, E: Error.

*No reports retrieved for the PT, however reports might be retrieved for other countries.

# Extracted means that we extracted the PT from MedDRA dictionary, the process explained in the methodology.
